# Women with Neck Pain on Long-Term Sick Leave—Approaches Used in the Return to Work Process: A Qualitative Study

**DOI:** 10.1007/s10926-016-9636-3

**Published:** 2016-03-05

**Authors:** Linda Ahlstrom, Lotta Dellve, Mats Hagberg, Karin Ahlberg

**Affiliations:** 1 Department of Public Health and Community Medicine, The Sahlgrenska Academy, University of Gothenburg, Medicinaregatan 16, P.O. Box 414, 405 30 Gothenburg, Sweden; 2 Faculty of Caring Science, Work Life and Social Welfare, University of Borås, Borås, Sweden; 3 Health and Care Sciences, The Sahlgrenska Academy, University of Gothenburg, Gothenburg, Sweden; 4 Department of Ergonomics, KTH - Royal Institute of Technology, School of Technology and Health, Stockholm, Sweden

**Keywords:** Rehabilitation, Work disability, Absenteeism, Grounded theory, Return to work

## Abstract

*Purpose* There are difficulties in the process of return to work (RTW) from long-term sick leave, both in general and regarding sick leave because of neck pain in particular. Neck pain is difficult to assess, problematic to rehabilitate, and hard to cure; and it is not always easy to decide whether the pain is work-related. The outcome of RTW could be dependent upon individuals’ approaches, defensive or offensive behaviors, and choices related to their self-efficacy. The aim of this study was to identify approaches used in the RTW process among women with neck pain on long-term sick leave from human service organizations. *Methods* This is a qualitative descriptive study based on grounded theory. A Swedish cohort of 207 women with a history of long-term sick leave with neck pain from human service organizations answered open-ended written questions at 0, 6, and 12 months, and 6 years; and 16 women were interviewed. *Results* Individuals expressed their coping approaches in terms of *fluctuating in work status over time*: either *as a strategy* or *as a consequence*. Periods of sick leave were interwoven with periods of work. The women were either *controlling the interaction* or *struggling in the interaction* with stakeholders. *Conclusions* Return to work outcomes may be improved if the fluctuating work status over time is taken into account in the design of rehabilitation efforts for women with a history of long-term sick leave and with chronical musculoskeletal conditions.

## Introduction

The most common causes of sick leave in Sweden are musculoskeletal disorders and mental health disorders; about 60 % of women on sick leave have one or both of these diagnoses. Women have a higher risk than men of long-term sickness, and working within municipal organizations and being a woman are the greatest risks for long-term sick leave [1]. Studies have reported difficulties in the process of return to work (RTW) from long-term sick leave, both in general [2–10] and regarding sick leave because of neck pain in particular [11–14]. Neck pain is difficult to assess, problematic to rehabilitate, and hard to cure; and it is not always easy to decide whether the pain is work-related [15, 16].

The individual’s own commitment seems of crucial importance for the outcome of the rehabilitation process, along with support from others and the ability to understand one’s own actions [17, 18]. Several studies have found that the individual’s own belief in their ability to work in the future is a strong predictor for actual RTW [3, 8, 19–21]. The outcome of RTW could be dependent upon individuals’ approaches, defensive or offensive behaviors, and choices related to their self-efficacy [22]. Moreover, the individual’s own influence and taking an active part in workplace adjustments are both important in enhancing RTW [19, 23, 24]. Thus, the rehabilitation process needs to be conducted collaboratively and with purposeful actions, using the individual’s resources as an asset. Other studies have pointed to the importance of involvement of the workplace, work adjustments, and supportive measures at the workplace [25]. Working demands and conditions ought to be adjusted in accordance with the individual’s needs, the nature of the work, and the attitudes of the management [26]. Positive perspectives from employers, as well as employers that act to adapt and adjust the workplace to enable individuals to perform and function at work, are related to improved RTW processes [27]. These preconditions should be present for the individual to be able to perform at work, to have the strength to act at their workplace, and to maintain an optimistic approach to work. These factors could also contribute to a respectful workplace climate for the individual [4, 26, 28].

Knowledge is limited regarding the experiences of rehabilitation measures on an individual level [29], and in particular how these interventions affect the individual, their actions, and the timing of the RTW process [30]. As women have a higher prevalence of long-term sick leave and permanent work disability [31–33] due to musculoskeletal and mental health disorders [1, 34], it is important to develop an understanding and gain deeper knowledge of the approaches being used in the RTW process for women. The aim of this study was to identify approaches used in the RTW process among women with neck pain on long-term sick leave from human service organizations.

## Method

### Study Design

The study was a longitudinal descriptive study with a constructing grounded theory approach described by Charmaz [35]. Written answers to open-ended questions from a cohort of women on long-term sick leave were used as data, and further complemented with qualitative in-depth intensive interviews with women still suffering from neck pain. This method was considered suitable to help understand the rehabilitation and RTW process among these women, focusing on identifying these women’s experiences from their own perspectives. Grounded theory explores social processes at different levels, such as the interaction between the individual and stakeholders of central importance for the RTW process. This method gives the researcher freedom to generate new concepts explaining human behavior and styles, and to generate an empirically grounded theory [35].

### Context

The women in this study were all part of the Swedish sick leave system. This system changed in 2008, with eligibility for sickness benefits being now focused more on the individual’s work ability rather than the disability that caused the inability to work. Prior to this, individuals could be on long-term sick leave for an unlimited time. The new system aimed for and required more collaboration and cooperation between all the stakeholders, and was intended to help motivate individuals to action and make them take more responsibility for the RTW process. In Sweden, there is no difference in individual compensation between diseases/disabilities caused by work and those not caused by work. Work ability needs to be decreased by at least 25 % for the individual to be covered, and it is possible to receive sickness benefits covering 25, 50, 75, or 100 % of the working degree.

In Sweden, the employer is responsible for the rehabilitation process in regard to RTW. During the first 90 days, the individual’s work ability is assessed in terms of whether they are able to work at their existing workplace and perform their existing work tasks, the employer should make necessary work place adjustments, trying to adapt work to the worker in order to enable the individual to return to work. From day 91 to day 180, the assessment is according to whether the individual would be able to work for the same employer but performing different work tasks, employer ought to accommodate more permanent change of work task. Finally, from day 181 to day 365, the individual’s work ability is assessed against the labor market as a whole, looking at whether there is any suitable work at all. The sick leave compensation can be extended under special circumstances, depending on the individual’s diagnosis. Following this, the individual can apply for sickness compensation, which is intended for those who will probably never be able to work full time due to illness, injury, or disability. Long-term sick leave (>60 days) in Sweden requires a certificate from a physician and agreement between the central stakeholders in the rehabilitation process: the Social Insurance Agency, the health care system, the employer, the individual’s union, and the Public Employment Service.

### Sample

**Fig. 1:**
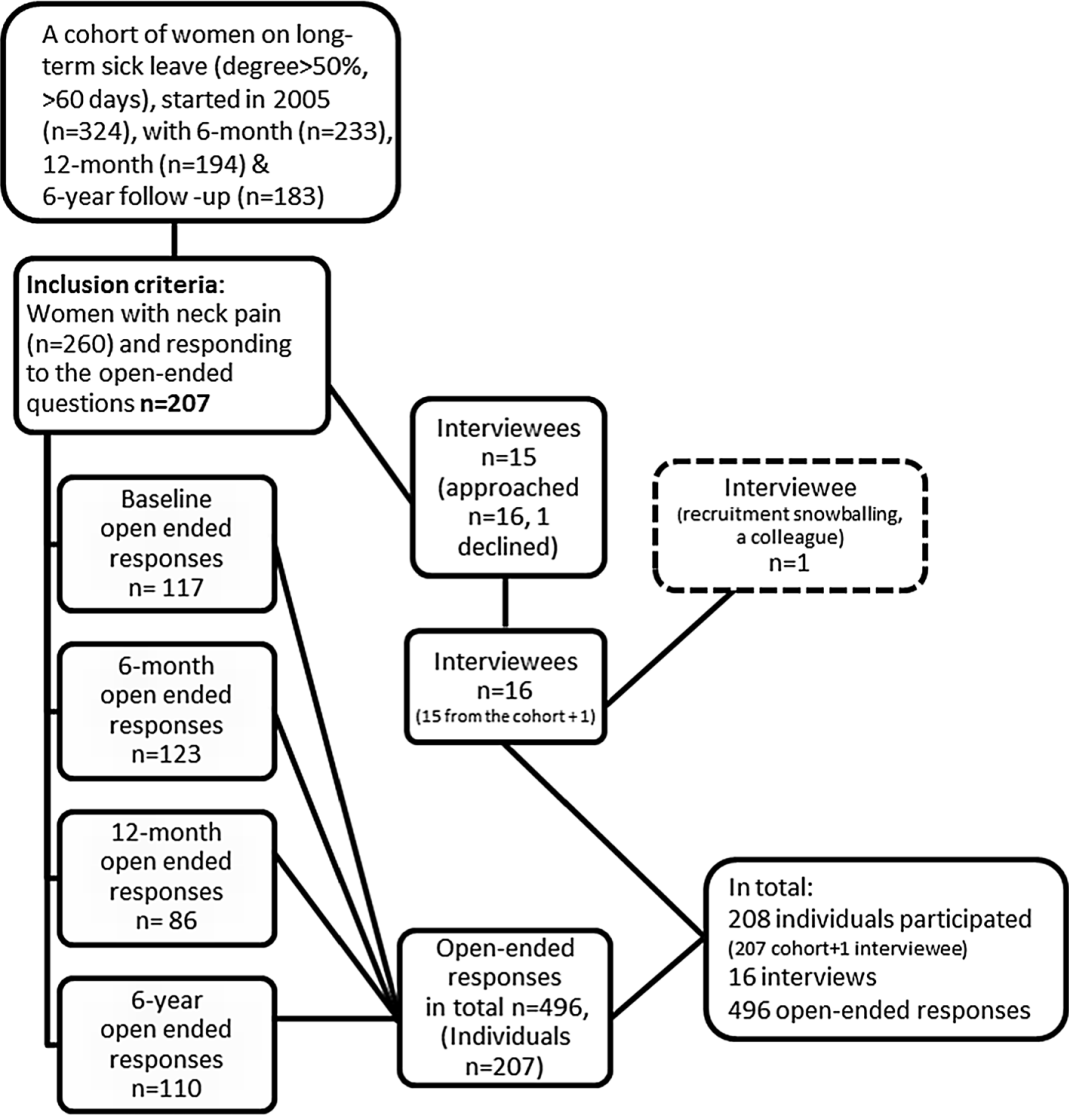
Flowchart of the participants in the study

A cohort of women (n = 324) working within human service organizations (HSOs) in Sweden and on long-term sick leave (>60 days, ≥50 %), at the time of recruitment, answered questionnaires on a number of occasions starting in August 2005 [25, 36] and later follow-ups after 6 months, 12 months, and 6 year. Quantitative studies have been reported earlier from this cohort [2, 6, 13, 36, 37], describing the cohort in detail. Data were collected from the answers to open-ended questions in this cohort study and complemented with 16 in-depth interviews. Of the interviewees 15 individuals were from this cohort and 1 additional interviewee were recruited through snowball sampling (Fig. 1). This study was approved by the Regional Ethical Review Board in Gothenburg (2005 and 2012).

#### Data Collection; Open-Ended Answers to the Questionnaires

Of all respondents in the cohort, 260 individuals (80 %) reported long-term/chronic pain in the neck region, and 207 of these (80 %) answered the open-ended questions; their answers were all included in this study. The inclusion criterion for the present study was having reported neck pain, at all measure points, with a score of at least 3 on the von Korff Pain Index [38]. This cut-off point is supported by the finding in a recent study that a score higher than 3.4 indicates pain that is moderate or severe and that interferes with functioning for patients with chronic musculoskeletal pain [39]. All of the individuals in our study reported neck pain, with an average score of 5 (SD 3). The open-ended questions from baseline, 6 months, 12 months, and 6 years were all used. The open-ended questions were concerned with facilitators for RTW, work adjustment, and experiences of sick leave and RTW. These questions were answered 496 times in total (177 participants at baseline, 123 at 6 months, 86 at 12 months, and 110 at 6 years).

#### Data Collection; Interviews

For the interviews, 16 cohort participants were approached, with a telephone call, using a strategic sampling of those who reported neck pain in the 6-year questionnaire in order to interview a variety of individuals with regard to age group, sick leave degree, and occupation. One of these individuals declined participation due to lack of time, but an additional interviewee was recruited through snowball sampling using the same inclusion criteria, to ensure variation in age. Hence, 16 interviews were conducted.

The interviews were conducted face to face by the first author (LA, PhD Student, Registered Nurse) at a time and place (the clinic or their home) chosen by the individual. The interviews typically lasted for an hour or an hour and half. Interviews were intensive, shaped, beneath the surface of normal dialogue [35], and recorded. The researcher (LA) took field notes after each interview, wrote memos and a short summary of the interview during the whole analytical process. Data from the in-depth interviews were collected using an interview guide, which was slightly modified during the interviewing process, the interviewer sometimes re-word, re-order or clarified the questions to further investigate topics introduced by the respondent, and being more focused along the analyzing process [40]. The guide focused on views, factors, and approaches used in the individuals’ rehabilitation processes, and set the following areas of questions: What are the interviewees’ own beliefs and suggestions about what could facilitate their own RTW? What are their own goals for rehabilitation and the RTW process? What are their own beliefs about their collaboration with stakeholders? Each interview was transcribed verbatim shortly after it took place.

#### Data; Open-Ended Answers and Interviews

A total of 208 individuals participated in this study (Fig. 1), with a mean age of 49 years among the cohort participants and 54 years among the interviewees (n = 207 from the cohort and n = 1 additional interviewee through snowball sampling). These women were working for some periods, alternating with being on sick leave for shorter and longer periods to different working degrees; so at the 6-year follow-up, the working degree among the participants ranged from 0 to 100 %. The 208 participants in this study group were checked and controlled for variety within the group concerning educational level, profession, diagnosis/disorder, and civil status, also taking into consideration the fact that they all worked in HSOs. The rationale for choosing women with neck pain was that this symptom was the most prevalent cause for long-term sick leave in Sweden at the time when the cohort was started in 2005. Another reason for selecting these individuals at the 6-year follow-up was that in order to meet the aim of the study, the participants needed to have long-term pain from a musculoskeletal disorder affecting work ability for a long period of time [15, 41, 42].

### Data Analyses

Throughout the analyses, the researchers aimed to follow the constructivist grounded theory approach according to Charmaz [35], using a back-and-forth process during the whole development of the study. The interviews and answers to the open-ended questions were read, and re-read; additionally the interviews were listened to again. Initial coding was conducted by the interviewer (LA), following constant comparison of the transcripts and memo notes as the data were collected. Open coding was performed, and throughout the analytical process categories were constructed when there were obvious relations. A category represented a unit of information composed of events, happenings, and instances. Coding was essential while transforming the raw data into constructions of the social process, and was performed by three of the authors (LA, LD, KA). Discrepancies in interpretation were discussed and re-examined among the researchers until consensus was achieved; to ensure consistency in application of the categories, an additional researcher was involved (MH). The researchers strove for an open mind while coding and analyzing the data. The resulting categories emerged during a process of constant comparison [43]. The researchers alternated between coding the open-ended questions and coding the interviews. When analyzing the open-ended questionnaire data, the researchers did not take the timing of the data collection into consideration. The first author conducted additional interviews one by one to allow further categories to emerge, using constant comparison throughout the analytical process. As the final interviews were analyzed, the same categories were seen rather than new categories, but the process continued until no new patterns emerged, indicating data saturation [35, 43]. The analytical process was a continuous one, meaning that the method was focused but flexible, and the data collection was more stringent in the end of the data collection. This enabled the researchers to make an in-depth exploration of the processes. The interview questions being more focused during the process. A qualitative data analysis software package (NVivo 10, QSR International) was used to manage the structure and sort the data.

## Results

### Fluctuating in Work Status Over Time (Core Category)

**Fig. 2:**
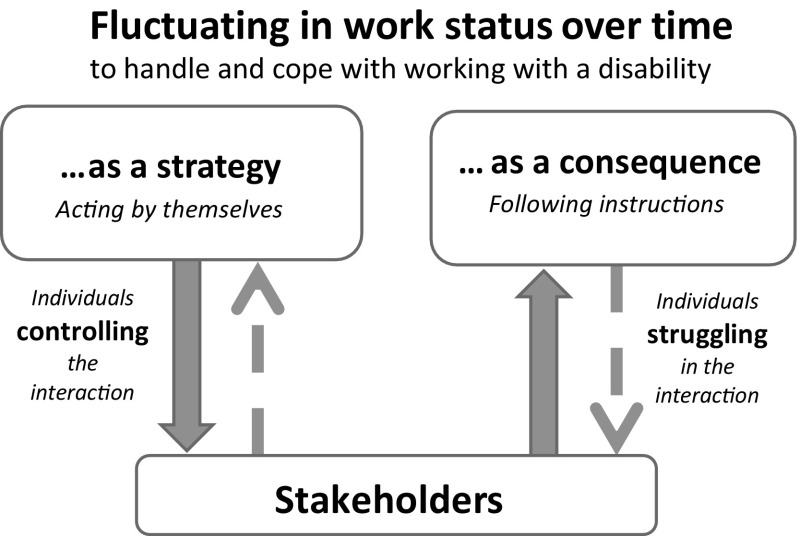
Model of approaches used in the process for return to work, described by women with neck pain on long-term sick leave working within human service organizations

The storyline revealed in this study described how these women with neck pain on long-term sick leave were *fluctuating in work status over time* (core category), fluctuation in working degree. They used different approaches towards this, in the rehabilitation process to cope with RTW (Fig. 2). There was a certain degree of “going in and out” of work participation, with periods of sick leave interleaved with periods of work. Fluctuating in work status over time was a way for the women to handle their health conditions in relation to the opportunities allowed them by their employer, the social security system, and the health care system. They expressed their desire to work, their goals for work, and their wishes for work, despite their disabilities. They communicated an optimistic mindset to be working, and they also described creativity, decisiveness, determination, and visionary thinking about their prospects of handling their work situation. Working is what people do, and working was what they wanted to do to the best of their abilities. They spoke about not being able to work full-time, as they preferably wanted to.

It’s no fun being on sick leave (full-time). Right now I’m trying to work part-time to a degree of 50 %. But I don’t know if it’ll be sustainable because sometimes it feels hopeless. After I’ve been working I have a lot of pain. But I don’t want to give up without trying, and I hope everything will be fine in the end (working full-time).

All the women had their own individual goals for success in RTW. They described two kinds of approaches for coping with RTW, both involving fluctuating in work status over time: *fluctuating in work status over time as a strategy* and *fluctuating in work status over time as a consequence*, the path to RTW was a complex one for both these approaches. During the process of RTW, the women experienced their interaction with stakeholders in diverse ways. Two main approaches were identified: either *controlling the interaction with stakeholders* or *struggling in the interaction with stakeholders*.

### Fluctuating in Work Status Over Time as a Strategy—Controlling the Interaction with Stakeholders

The women using *fluctuating in work status over time as a strategy* were creatively enterprising and took responsibility, drove things forward, and worked hard to achieve success in their RTW process; they held control in their own hands, they were acting and making their own decisions. The participants used the strategy of fluctuating in work status over time to allow them to cope with working life and make timely adjustments to their RTW status. (Table 1, Quotations 1–7). They focused on what they as individuals were capable of doing at work, and their own working abilities. They found their own solutions and their own ways to cope with work; they did not want to ever again reach the point of returning to full-time sick leave. They wanted to survive, and had come to the decision that they could not manage full-time work all the time. They knew how the sick leave system worked according to the social security system and employment roles, allowing them to be off sick for a certain number of days, a specific number of times per year. They strategically planned their vacation in combination with this to help themselves cope and function through the working year. To keep a good work-life balance, some chose to work less and earn less. Even though working less resulted in the inability to afford extras, it gave them the strength and spare time to do something other than working. They realized and appreciated the importance of having a life outside work (work-life balance), they had learnt to set their boundaries; they had found their work assets and limitations. Most of them said that they had been learning their own limitations at work and how to say no at work. These individuals were taking the helm, saying that only they could help themselves.

**Table 1:** Quotations exemplifying the categories for women on long-term sick leave with neck pain

Quotation number	Category	Source^a^	Quotation translated to English
1	Fluctuating in work status over time as a strategy	Interview (Holly)	So then I took sick leave for a week, and then I went to a doctor who gave me a sick leave certificate, and then I started working again
2	Fluctuating in work status over time as a strategy	Interview (Ida)	I went back to the doctor again, now I was tired and worn out, I still had pain… couldn’t perform my duties at work, it was very hard, so maybe the doctor could write me a sick leave certificate for two weeks, so I could recover and get some strength back… to cope again
3	Fluctuating in work status over time as a strategy	Cohort (baseline)	I don’t think I should be working full time. I need time for walking, water aerobics, and other exercise
4	Fluctuating in work status over time as a strategy	Cohort (6 months)	I’ll last maybe half a year (100 % working). I’ve only been resting (100 % sick leave) to cope with “a new turn”, no changes in my work situation
5	Fluctuating in work status over time as a strategy	Cohort (12 months)	I’m now working 75 %. I’m very tired after a full day’s work, and I need 25 % sick leave for training and relaxation.
6	Fluctuating in work status over time as a strategy	Cohort (12 months)	Full-time sick leave now, I’ve worked half time during this year. I’ve managed 3–4 weeks. Then I had to take vacation to be able to cope with working again. I’ve now come to the conclusion that it’s not the way to handle the problem
7	Fluctuating in work status over time as a strategy	Cohort (6 years)	I had to change, and so I ended up not being bothered if I don’t always do my work properly. When I realize it’s not possible to continue working anymore, I make boundaries and I take care of myself, I go home and leave my work tasks behind
8	Controlling the interaction with stakeholders	Interview (Ella)	And things get delayed; it takes a long time before anything gets started at all, so I’m the one who’s proactive. I’m constantly driving things forward and I’m the one who’s taken action… but I have such a strong will and I’ll fix it and I’ll manage
9	Controlling the interaction with stakeholders	Interview (Ella)	I demanded to see Occupational Health and at first my supervisor said no. And then I said “I have to do it,” so then it took about a month before I got there
10	Controlling the interaction with stakeholders	Cohort (6 months)	Now I’m better again and I’ve finally been able to increase my working hours (after some work adjustments and flexible working hours) and it feels great
11	Controlling the interaction with stakeholders	Cohort (6 months)	I work by myself a lot, and have learned to say no to work tasks many times per week
12	Controlling the interaction with stakeholders	Cohort (12 months)	The rehabilitation and tailored interventions were in consultation and collaboration with my employer and the Social Insurance Agency
13	Fluctuating in work status over time as a consequence	Interview (Ida)	I feel like I had no say in the matter, no chance to choose, I had no choice, so then I did work that I shouldn’t have done. No, that wasn’t good
14	Fluctuating in work status over time as a consequence	Interview (Josefine)	In the beginning I didn’t know what I needed to do to be on sick leave. I was told I might need to take sick leave now… I don’t know what the purpose was to take sick leave. But the doctor decided it was good for me
15	Fluctuating in work status over time as a consequence	Cohort (baseline)	I’m back at the same workplace that I was burned out at; I don’t think it’ll last very long. But I haven’t received any alternative from my employer
16	Fluctuating in work status over time as a consequence	Cohort (12 months)	Now I’ve completed 75 % of the rehabilitation program. Now I’m on 50 % sick leave and 50 % working. I’m waiting for the outcome of their (the Social Insurance Agency) investigation about sickness benefits
17	Fluctuating in work status over time as a consequence	Cohort (6 years)	I have received preventive sick leave to let me work out at the gym
18	Struggling in the interaction with stakeholders	Interview (Felicia)	It sounded so negative and weak and wimpy, my condition, so I just said my symptoms, not what I thought it was. And then the doctor asked a lot of follow-up questions and then the doctor wrote a list of what I should do. And so I did it
19	Struggling in the interaction with stakeholders	Interview (Greta)	It didn’t feel like you’re worth a fair chance, or, what should I say, you should just be grateful and accept it. So I thought it was very difficult then to be involved
20	Struggling in the interaction with stakeholders	Interview (Greta)	Several times during this rehabilitation process they called from SIA [the Swedish Social Insurance Agency] and asked when I could start to work. It was like; can you come and show yourself? Of course I can, but I’m disabled and I can’t walk, if they can arrange transportation then I can come. They have no control, really
21	Struggling in the interaction with stakeholders	Cohort (12 months)	I’m on sick leave because I was transferred to night shifts. It’s related to cost savings at work that I have to work night shifts again. I have a medical certificate stating that I shouldn’t work night shifts. I worked night shifts when I was on long-term sick leave, and that didn’t work
22	Struggling in the interaction with stakeholders	Cohort (12 months)	It doesn’t feel good at all to return to work, because I was forced back to work. Why? Because I don’t feel well, I feel worse now. The Social Insurance Agency forced me to return to work
23	Struggling in the interaction with stakeholders	Cohort (6 years)	I was repeatedly doing work tasks that I had no ability for because of my illness. The employer/supervisor did not listen or take my disability seriously

The core category was fluctuating in work status over time

^a^Interviewees’ names are fictional

These women expressed their RTW as their own responsibility, and they understood that they had to act themselves in the rehabilitation process for RTW; they were *controlling the interaction with stakeholders*. They had the inner strength to set their own goals, and to express their own will and needs to the stakeholders. They realized that they had to change their approaches for coping with work, in order to build their self-confidence. After RTW, these women adjusted their own working demands to allow them function at work, or ensured they changed work position in order to be certain that they could manage the work on a daily basis. They had come to terms with setting their own limits on what they were capable of, and imposing requirements on their employers (Table 1, Quotations 8–12). They had also accepted the idea that no one else would fix their problems, and they would have to dare to act themselves.

These women had been constantly managing and taking action themselves through the rehabilitation process, and controlling the interaction with stakeholders. In due time, they found their own solutions and their own way, and resolved their own difficulties in the process of RTW, informing and instructing the stakeholders. They had not relied on the stakeholders, but rather themselves when it came to RTW; they had not given up, but had remained clear about their direction and their goals. These individuals had set the standards when interacting and collaborating with stakeholders. These individuals perceived that their supervisors had understood the situation and supported their rehabilitation actions, taking the attitude that their employers wanted them to RTW. They felt that all stakeholders had to work as a team, and made sure everyone in the team was working in their desired direction. The women knew what kind of support they needed in their rehabilitation process, for example from their medical center and physicians; if they were not satisfied, they changed facility. The women themselves decided when it was time to RTW, and informed the stakeholders when or to what extent they required sick leave or not.

### Fluctuating in Work Status Over Time as a Consequence—Struggling in the Interaction with Stakeholders

Women with *fluctuating in work status over time as a consequence* were those who unquestioningly were taking instructions from stakeholders, they waited for and obediently followed orders without resistance, and the individuals thus had to handle different stakeholders’ information in order to cope. The women thought that the stakeholders acted in a way that allowed them to cope with their daily lives and to have a work-life balance (Table 1, Quotations 13–17). They went in the direction they were told by the stakeholders, not knowing where this would lead, or even if it would lead anywhere. They took instructions and waited for orders to be given in the RTW process. They felt that their RTW was their employers’ responsibility.

Different stakeholders had different goals in relation to the individuals’ working degree, and did not always collaborate with each other; this often made things confusing for the individuals who wanted to follow all the instructions given. The women believed that the stakeholders wanted to prevent stress and pain for them, and that the stakeholders realized they could often only manage part-time work. They expressed a belief that the stakeholders were emphasizing the individuals’ need for physical exercise and different training programs, and understanding that the women needed compensatory time for doing these rehabilitation measures, resulting in them being on part-time sick leave. Thus, the women were complying with rehabilitation treatment. The different stakeholders were perceived as uncoordinated, not adapting to the individuals’ needs, and lacking in respect for them as individuals. The women themselves had a lot of respect towards the other stakeholders and societal systems, and were compliant with these other actors’ directives in the rehabilitation process. They complied with the rehabilitation activities and working degree suggested by the stakeholders, even though they knew this was doomed to fail. They followed the fluctuating in work status over time pattern in order to survive in the social security system of RTW. They wanted to remain in the social security system, as employment status was taken into consideration and they needed to earn their living. They did not seek contact with the stakeholders, but their employers had the obligation to make contact with them. The participants felt it was better to work for a little bit than not at all. They expressed the importance of social coherence at the workplace, and being part of a group, and so they managed to work for at least a couple of hours. Reasons for working included the importance of socializing at the workplace; they had the desire to work and be part of a community.

These women were being responsible and compliant, and were easily managed and controlled by the stakeholders; the individuals were *struggling in the interaction with stakeholders*. As a consequence, they felt they were being pacified in their RTW process and did not believe their views were important. They felt they were not being listened to and that the other stakeholders were not interested in their opinions. They relied on the other stakeholders’ expertise and knowledge (Table 1, Quotations 18–23). The women often got stuck in this process; they lost their desire and energy, and became listless. Many of them had followed instructions to try out several different workplaces, but had failed, and then became indifferent and apathetic. The consequence of this was that the women lacked a focus on themselves, and missed being an active part in the jointly planned rehabilitation process.

These women described how their leaders and colleagues acted for them in the rehabilitation process; they had the feeling that their wishes were not being listened to, but in fact they had not informed the stakeholders about their wishes. They expressed how the focus in the rehabilitation process had been placed on their limitations, and on what they could not do; their employers were focusing on excluding work tasks rather than working the other way around to discover possibilities for them to perform tasks at the workplace. The milestones within the rehabilitation process were not synchronized among the stakeholders, and definitely did not involve the individuals who were work-disabled. Further, the women said that conversations during appointments with their physicians were almost entirely concerned with whether they needed sick leave or not, with the physician making the decision. Some of them did not even know the reason for their being on sick leave and unable to work; they stated their physician had told them to go on sick leave. Often they did not know what to do from a rehabilitation perspective while on sick leave; mostly, they just wanted the time to pass, with no rehabilitation goals for the sick leave period. They had the impression that it was all about emergency solutions, not about the rehabilitation process for RTW. Their experience was that other stakeholders did not meet or even see their need to RTW, and they were viewed as just another case.

## Discussion

This study highlights women’s desire to cope with and handle their rehabilitation process and RTW through the approach of fluctuating in work status over time, either as a strategy or as a consequence. These approaches were related to different ways of interacting with stakeholders involved in their RTW process; the women either controlled the interaction or struggled in the interaction. Present findings will be discussed in relation to the theory of self-efficacy [22] and the Sherbrooke model of RTW [29]. In the rehabilitation process for RTW, individuals are affected by different contexts, such as the workplace system, the social security system, the health care system, and the personal system (resources and coping mechanisms). The focus in this study was on these systems from the perspective of the individuals with chronical or long-term disabilities, especially those with neck pain.

Self-efficacy has been highlighted as essential to the success of the RTW process [44, 45], and as a predictor for RTW as recently been shown in Brouwer et al. study among individuals with upper extremity and back musculoskeletal disorders [46]. Self-efficacy is belief in one’s own ability to perform actions; the greater an individual’s confidence in themself, the more likely they will initiate and continue an activity that results in a positive outcome [22, 47]. An individual with a high degree of self-efficacy often chooses more challenging tasks and has a higher capacity for endurance than people with lower levels of self-efficacy. Self-efficacy is related to both self-rated physical work ability and mental work ability [48]. Degree of self-efficacy affects the coping strategies that an individual uses; individuals with high self-efficacy are more likely to see obstacles as challenges, which makes them more solution-oriented, produces greater effort to reach their set goals, and makes them less likely to give up. From our results, we can anticipate that individuals who struggle in the interaction with stakeholders will, conversely, experience goals as a hindrance. An individual’s degree of self-efficacy has previously been shown to affect their opportunity for coping strategies such as planning, humor, acceptance, and accommodating to their situation [49]; could the fluctuating in work status over time pattern be seen as the result of this. Research shows that the application of the theory of self-efficacy in clinical practice is successful in promoting the rehabilitation process for individuals, and decreasing disability and pain; this research also addresses the connection between physical and mental health [50]. These results are likely transferable to our context, the approaches of the women on long-term sick leave described in our study could be connected to a lower or higher self-efficacy. Reflecting the “I can do it approach”, or high self-efficacy, representing the women using fluctuating in work status over time as a strategy. Conversely, it is possible that the women with low self-efficacy were those who did not see themselves as being able to successfully carry out the stakeholders’ suggested actions. These women were following instructions that were not appropriate, involving unadjusted rehabilitation measures and thoughtless RTW actions. They could foresee they would not be able to do these work functions, as they lacked the work ability, skills, and competencies, yet they did not dare to express this information and did their utmost to fulfill these obligations despite knowing it would not lead to a sustainable RTW. Thus, it can be argued that higher self-efficacy could strengthen women in using fluctuating in work status over time as a strategy and controlling the interaction in the collaboration with stakeholders. In contrast, for women with weak self-efficacy, fluctuating in work status over time would more likely be a consequence and a sign of struggling in the interaction with stakeholders. On the other hand conclusions from a Danish cohort study indicated that lower self-efficacy can be a result of the sick leave or disorder itself, via its effect on the individual’s position in the labor market [51]. While other studies have argued for socioeconomic explanations for self-efficacy, suggesting that higher self-efficacy is associated with higher socioeconomic status [45], it can be debated whether the women in the present study would have lower self-efficacy due to their work within HSOs. We tend to agree with Labriola et al. [51] that individuals perceived self-efficacy are related to the RTW process, as well as most likely the fluctuating in work status over time pattern. Self-efficacy can have an important meaning when it comes to understanding the women’s approaches and offering support to them in the RTW process.

Ståhl et al. [52] have previously mentioned the problem of individuals who have been assessed as work-disabled according to certain work activities but are forced into labor market integration anyway. These actions have been identified as related to the change in Swedish sick leave regulations, demanding a work ability assessment. The consequence of these rehabilitation activities might impact women’s self-efficacy, and as a result they might have developed different approaches in their interaction with stakeholders. Individuals’ self-efficacy will further be affected in diverse directions through success in rehabilitation activities and RTW. To enhance this hopefully positive direction, stakeholders could try to shape their expectations to fit individuals’ capacities in relation to work tasks, when this is possible [44], and to interact and have a discussion with the individuals about their plans and goals. This interplay is of importance in creating supportive organizations which can facilitate increased work ability and RTW for the individual [2, 29, 53].

The RTW process involves the workplace/employer, the social security system, the health care system, and the individual’s personal system of resources and coping mechanisms [54]. Incorporating the Sherbrooke model into the rehabilitation process means taking into account and involving all stakeholders, most importantly the individual undergoing the rehabilitation process to RTW. All the arenas involved in the RTW process [29] were clearly mentioned in the interviews analyzed in this study, and the interviewees also spoke about how these systems affected their rehabilitation and RTW process. The social security systems and workplace systems all strictly followed their formal requirements, often regardless of the individual’s needs and wishes. An explanation for this, as pointed out by Ståhl et al. [52], is that the employees at the Swedish Social Insurance Agency themselves experience a lack of competence to assess work ability, and there is also a lack of collaboration between stakeholders. In order to be able to enhance and improve RTW for these women, it is essential for stakeholders to use similar concepts, to try to have a shared understanding, and to work towards the same goals. It is of great importance to also share these targets with the individuals who are returning to work, as an awareness of the stakeholders’ competing goals can help promote their welfare [55]. Further a recent Swedish study reported that stakeholders, specifically the social security stakeholders, have become gradually more passive since the social security system changed in Sweden in 2008, to number of days on sick leave being limited to one year. The aim of that reform was to involve all stakeholders in the process. The individuals themselves have been given more responsibility [56], but they have not been prepared to take this role and do not know how to act. The consequence of this could be an inability to optimize the rehabilitation process for the individual, which in turn leads to the individual feeling that they are not being listened to. The participants in the present study expressed the feeling of being trapped in the rehabilitation arena, themselves not knowing the solution to their situation, and not being aware of the plan of the RTW process. Earlier research into women’s approaches to RTW found that women were affected by very diverse perspectives on what factors enhance recovery and promote RTW [57]. The women and stakeholders could benefit from being aware that the rehabilitation process is complex and dynamic. The rehabilitation process could benefit from being mutual, interactive, and collaborative between all stakeholders involved, and it is dependent on both individual and societal/environmental aspects [58]. A recent Swedish qualitative study of stakeholders’ attitudes and focus in rehabilitation meetings revealed that there is an unequal balance of power involved in these meetings, with employers having the leading position. The employers were in charge of workplace adjustments, and so their opinions and cooperation repeatedly decided individuals’ possibilities for RTW [27]. Therefore being aware of and highlighting this power balance might be a way to reduce the occurrence of negative experiences. Noteworthy is that the Swedish social security system will now (2016) change again to become almost unlimited, when it comes to days on sick leave, for the individual, thus the interaction and the relationship between all stakeholders involved will be even more crucial.

From a societal perspective, the Swedish workforce includes an increasing number of individuals with chronic or long-term diseases and disorders; as in other western countries, and in the future people will have to continue working to higher ages. Thus there is a need to focus on how to improve the situation for individuals returning to work [59], despite their chronical conditions, and how to enable them to stay at work. Long-term sick leave and long-term disabilities is a public health problem, and one which causes a great deal of distress both for the individual and for society [60]. Returning to work from long-term sick leave requires the ability to cope throughout the RTW process, as highlighted by the different approaches used by the women in the present study. The presence of well-trained and well-educated teams to plan and coordinate the RTW process, as well as an awareness of individuals’ different approaches, might therefore be of great importance to understand and enhance the process [61]. Teams ought to work within the areas of communication and conflict resolution for the individual at the work place, and to have competence in fostering interpersonal relationships and communication [62]. Workplace interventions and supportive conditions at work are essential for success in the RTW process for individuals on long-term sick leave [25], and especially good quality of leadership are crucial for increased RTW [2]. If employers could obtain positive outcomes from accepting and accommodating fluctuating in work status over time for the individual with a disability, these employers may then cooperate in allowing the individual to fluctuate in working degree. This could be one way to boost self-confidence. Making this way of working more acceptable could help disabled people to manage work; this might work in different work settings and in different contexts, but not everywhere.

There needs to be a discussion about the goals of work participation and working degree for individuals with a chronical condition or a disability/disorder that might affect their work ability [63, 64]. One likely solution for these individuals would be for them to choose fluctuating in work status over time, and for society to accept this behavior. The different stakeholders involved could try to collaborate and match the individual’s needs, and have shared goals and a mutually-defined “good outcome” of the RTW. There is a desire from the government side for the stakeholders to express, articulate, operationalize, and evaluate the goal of RTW for the individual [55]; before this is done, it could be difficult for them to work together and collaborate towards the shared goals. If these women can be made to feel part of the workforce, with a sense of belonging, there would be benefit both for society and for the individuals. Being aware of and open-minded about individuals’ different approaches to fluctuating in work status over time, while understanding individuals’ ways of coping with interactions with stakeholders, offers a respectful way to intervene in and appropriately guide the RTW process. Further, it is a challenge for individuals previously on long-term sick leave to re-enter the labor market; one reason for this is that doing so carries a high risk that they will have to return to long-term sick leave, and another is that there is no margin of maneuver at the workplace [58].

Individuals’ resources and strengths can be better used in order to optimize the rehabilitation process. Some women in our study made their best of their preconditions by controlling the situation, while others struggled with the interaction with stakeholders. Information, involvement, and collaboration are essential for individuals’ commitment to the RTW process, and for the feeling that they are involved in the process and supported by the stakeholders and thus able to take deliberate actions of their own choice [22]. The women who were controlling the interaction in the rehabilitation process had chosen their own direction for RTW; from a stakeholder’s perspective, this could either make the rehabilitation more difficult or make it easier. For these women, the stakeholders needed to make demands, challenge them, and confront them, while at the same time being more supportive. It is possible that there are more obstacles during the rehabilitation process when the individual is unclear about their wishes and goals in the rehabilitation process, and the stakeholders are uncertain of the course. The results indicate that an important starting point is the individual’s story, including their motives and views on the rehabilitation process for RTW; from there, a mutual rehabilitation plan could be created incorporating goals and strategies for short- and long-term follow-up.

From an employer perspective, it is likely more productive to have an individual at work who has the ability to perform, rather than having an individual who is present but feels incapable of working. Adjusting the working hours and days can enable the individual to perform at work and to be productive during working time, and so in order to reduce waste it might be a good solution to let the individual decide their own working hours and days, when possible [65]. The rationalization used at workplaces today has been proven to have a worker health effect; it is a risk factor for musculoskeletal and mental health disabilities. This knowledge could encourage employers to embrace the concept of allowing the individual to choose fluctuating in work status over time when plausible, and to highlight the fact that sustainable production systems must involve the employee. Future sustainability might involve more flexible working hours and working days to accommodate all workers, disabled or not, creating a balance between production and worker well-being, and allowing individuals to remain in work.

It has also been demonstrated—and could be important for organizations to be aware - that employees with high self-efficacy are more willing to accept change, better able to adapt to change [66], and more committed to their workplace; they feel competent, and in some cases believe they would not succeed as well at a different workplace [67]. Additionally there is evidence that implementing interventions addressing organizational culture, having supportive policies, and providing educational interventions to improve self-efficacy offer a successful way to enhance RTW [68].

### Ethical Considerations

It could be demanding for individuals to have to rethink what had happened during their rehabilitation process, and to realize that matters could have been managed differently. There has been debate over whether the approach of interviewing individuals meets the requirements for good practice; will data emerge and bring valuable information about the rehabilitation process, or will the researcher only have an impact on individuals? The researcher indirectly makes a judgment on the data when considering whether the findings will be beneficial. Bearing in mind the ethical issues, when collecting personal sensitive interview data from an individual, the researcher needs to be sensitive to where the individual’s comfort zone is, and should not push the individual to share information they did not intend to share. Often it could be better if the researcher just listens to what the individual is telling them [35]. Revealing sensitive information could be risky and emotionally difficult, so the individual might (consciously or unconsciously) fail to share these thoughts in the interview.

### Methodological Discussion

From the onset of the study, the novice researcher began the data collecting phase and started the initial coding, supported by the senior r*e*searcher. Transcripts, codes, and memo notes were discussed and revised with the wider research team (LA, LD, MH, KA) to reach consensus, and tentative categories emerged through focused coding. The researchers aimed to achieve high levels of quality while being extremely thorough, careful, trustworthy, and strict throughout the analytical process to ensure that the interpretation was grounded in the data [69, 70]. The method of constructing grounded theory argues that recording and transcribing interviews could be beneficial in regards to hearing participants’ voices, tones, tempo, and pauses [35], and also to remember questions asked by the researcher: the construct of the interview. We are, however, aware that there are other standpoints on this issue. Recordings also made it possible for the other researchers in the group to listen to the interviews. In order to increase the trustworthiness and rigor of the analysis, both individual and collective analyses were conducted. This process was enhanced by all the researchers having access to all the data and codes, and the presence of a well-developed structure enabling comparisons and flexibility in coding due to the use of a computer-assisted qualitative data analysis software program, Nvivo [71]. The transparency of the research project was ensured by using this very structured way of working through the data with the grounded theory approach [72].

Important bias factor could be the possibility that the individuals will present a favorable image of themselves the social desirable responding, but then again considering the questionnaires being anonymous this factor hopefully is not affecting individuals’ responses. Further respondents were informed aware researcher had no connection with the employer or the social security system. The researchers are aware that in this analytic process we closely engage with the participants and therefore are unable to completely avoid personal bias [40].

Some scholars state that the researcher should enter the research arena without doing any research in advance within the subject, without taking any theoretical perspectives, and trusting in emergence [43]. This can be debated, as some have asked whether a literature review can be used to improve, rather than hinder, the development of theory [73]. However, it is best for the researcher to encounter the data with no preconceived ideas, and to be able to analyze the data without having a biased interpretation. As pointed out by Charmaz, it is impossible to interpret data in a completely unbiased way, as everyone has their own knowledge and experiences that cannot be ignored. Still, the researcher ought to see this as an advantage; the researcher is a social being within a social process [35]. The data will be looked upon from the researcher’s perspective, and so researchers must be aware of their own previous knowledge and remember that theirs is just one point of view, and not the only right answer or the only possible interpretation of the data. We chose to do a literature review in parallel with our analysis, and as our theory emerged we considered other researchers’ standpoints as well.

## Conclusions

Women on long-term sick leave with neck pain were *fluctuating in work status over time* to cope with their work. Two different approaches were identified: *fluctuating in work status over time as a strategy* and *fluctuating in work status over time as a consequence*. The women perceived their collaborating interaction with stakeholders as either *controlling the interaction with stakeholders* or *struggling in the interaction with stakeholders*. Return to work outcomes may be improved if the fluctuating work status over time is taken into account in the design of rehabilitation efforts for women with a history of long-term sick leave and with chronical musculoskeletal conditions.
